# Mr. Vandier, on the Vitality of the Blood

**Published:** 1801-12

**Authors:** F. N. Vandier

**Affiliations:** Great Mary le Bone Street


### To Dr. BATTY.

Dear Sir,

I Read the following Extract in the third Number of the Décade Philofophique, An. x. As it appears to me highly interefting, and as my engagements prevent me from repeating the experiment, I fend you a tranflation of it, which is entirely at your difpofal. I am, &c.

F. N. VANDIER.

*Great Mary le Bone Street, Nov. 13, 1801.*

*Extract*

*Extract of a letter to Professor Volta, on Animal Electricity, by J. Tourdes, Professor in the School of Medicine at Strasbourg.*

"Our intimacy, and the friendſhip of which you gave me ſo many proofs during my ſtay in Italy, prompt me to communicate to you the reſult of an experiment which ſeems to me to reſolve one of the moſt controverted problems of Phyſiology; I mean the *vitality of the blood.* That liquid, deprived of its aqueous humour, lymph, &c. and reduced merely to the fibrous part, was ſubjected to your Galvanic or rather electric apparatus, (for your laſt reſearches prove in an incontrovertible manner the identity of the Galvanic and electric fluids.)* The temperature was about 30° of Reaumur. It preſented oſcillations, a fluttering and palpitation, analogous to thoſe which are experienced by the fleſhy parts of recently ſlaughtered animals, *a double motion of contraction and dilatation*, very eaſily diſcernible by means of a magnifying glaſs, and which is a characteriſtic property of the vital principle, (or vis vitæ) peculiar to the muſcles, cellular texture, &c. &c."

* This identity has, I underſtand, been proved in London, in a very ingenious manner, by Dr. Wollaſton, Mr. Carliſle, &c.

